# Role of systemic corticosteroids in orbital cellulitis: a meta-analysis and literature review^☆^

**DOI:** 10.1016/j.bjorl.2021.02.003

**Published:** 2021-03-06

**Authors:** Boo-Young Kim, Jung Ho Bae

**Affiliations:** Ewha Womans University of Korea, School of Medicine, Department of Otorhinolaryngology, Seoul, Republic of Korea

**Keywords:** Orbital cellulitis, Infection, Steroid, Hospital stays, Surgical drainage

## Abstract

**Introduction:**

The standard management of orbital cellulitis is to administer a combination of intravenous broad-spectrum antibiotics along with treatment of associated sinusitis.

**Objective:**

The purpose of this study was to evaluate whether the addition of corticosteroids could lead to earlier resolution of inflammation and improve disease outcome.

**Methods:**

We independently searched five databases (PubMed, SCOPUS, Embase, the Web of Science, and the Cochrane database) for studies published as recent as December 2019. Of the included studies, we reviewed orbital cellulitis and disease morbidity through lengths of hospitalization, incidence of surgical drainage, periorbital edema, vision, levels or C-reactive protein, and serum WBC levels in order to focus on comparing steroid with antibiotics treated group and only antibiotics treated group.

**Results:**

Lengths of hospitalization after admission as diagnosed as orbital cellulitis (SMD = −4.02 [−7.93; −0.12], *p*-value = 0.04, I^2^ = 96.9%) decrease in steroid with antibiotics treated group compared to antibiotics only treated group. Incidence of surgical drainage (OR = 0.78 [0.27; 2.23], *p*-value = 0.64, I^2^ = 0.0%) was lower in the steroid with antibiotics treated group compared to the antibiotics only treated group.

**Conclusion:**

Use of systemic steroids as an adjunct to systemic antibiotic therapy for orbital cellulitis may decrease orbital inflammation with a low risk of exacerbating infection. Based on our analysis, we concluded that early use of steroids for a short period can help shorten hospitalization days and prevent inflammation progression.

## Introduction

Orbital cellulitis infects the orbital tissues, with chemosis and periorbital swelling accompanied by limited eye movements, proptosis, and on occasion, decreased visual acuity.^1^ The most common cause is rhinosinusitis extending into the orbit, especially when the ethmoidal sinuses are involved.^1^ The standard management of orbital cellulitis is to start a combination of intravenous broad-spectrum antibiotics along with treatment of associated sinusitis, and surgical drainage of the abscess is performed, when necessary. Although this effectively controls the infective component, inflammation sequelae may persist for weeks to months after infection.^2^

Studies have shown that systemic corticosteroids may be beneficial in treating acute rhinosinusitis, but clinical data on their potential role in orbital cellulitis are scarce. Acute orbital cellulitis attributable to bacterial infection is associated with diffuse edema of the orbital tissues with infiltration by inflammatory cells.^3^, ^4^, ^5^ As the orbit represents a nearly closed compartment with limited space, elevated orbital pressure as a result of inflammation is the cause of visual impairment in some cases of orbital cellulitis. By reducing edema and cell migration and inhibiting the toxic effects of cytokines and other mediators, corticosteroids can decrease the compression of orbital structures. Moreover, by decreasing fibroblast proliferation, corticosteroids can reduce scarring and potential long-term sequelae.^2^ The purpose of this study was to evaluate whether the addition of corticosteroids to indicated antibiotic therapy could lead to earlier resolution of inflammation and improve disease outcome.

## Methods

### Literature search strategy

Clinical studies published up till December 2019 were identified from PubMed, SCOPUS, Embase, the Web of Science, and the Cochrane Central Register of Controlled Trials. The search terms were as follows: “orbital cellulitis”, “corticosteroid,” “steroid”, “disturbance”, “sinusitis”, “complication” and “ptosis”. Only studies written in English were included. References of searched studies were identified to ensure that no related studies were overlooked. Two reviewers, working independently, scanned all abstracts and titles for candidate studies and removed studies that were not associated with orbital cellulitis and steroid treatment.

### Selection and exclusion criteria

All studies with patients with suspected orbital infection with or without sinusitis were searched. The studies were conducted based on the medical records of all patients reported from hospitals with the diagnosis of orbital cellulitis. Data collected from patient files included demographic parameters (age, nationality, immunization status), clinical parameters (the time of disease onset, clinical symptoms, examination findings, source of infection), laboratory and radiological findings (leukocyte counts and C-Reactive Protein (CRP) levels, microbiologic studies, radiologic findings, and CT scan), required treatment (steroid, antibiotics, surgical procedures), complications, and followup. For statistical analysis, the patients were divided into two groups: orbital cellulitis with both steroid and antibiotics treated group and orbital cellulitis with antibiotics only treated group. Patients were considered to have orbital cellulitis if they had signs of orbital involvement (photophobia, proptosis, painful extraocular motion, ophthalmoplegia, visual impairment, or chemosis), and confirmation of post-septal involvement was performed by computed tomography (CT).

**Figure 1:**
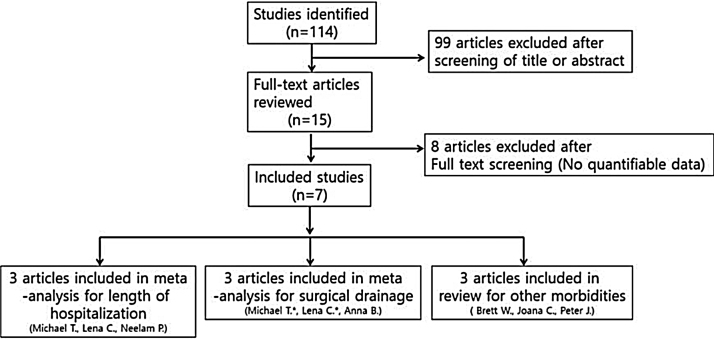
Diagram of the selection of studies for meta-analysis (*Data means repeated journal).

Studies were not considered adequate when including patients diagnosed with initial orbital abscess formation, orbital inflammatory syndrome presenting with intracranial abscess/infection, immunocompromised patients, and patients with a contraindication to receiving systemic steroids, or if reports were duplicated. In addition, studies were excluded from the analysis if clinical outcomes were not clearly described with quantifiable data or if it was not possible to evaluate the data from the described results. The search strategy summarizes the screening of studies chosen for meta-analysis (Fig. 1).

### Data extraction and risk of bias assessment

Data from studies were extracted with standardized forms and identified by two reviewers working separately. Outcomes for analysis included orbital cellulitis and disease morbidity to focus on comparing steroid with antibiotics treated group and antibiotics only treated group. From the included studies, we extracted the following data: the number of patients, length of hospitalization, incidence of surgical drainage, pain, periorbital edema, vision, levels or C-reactive protein, and serum white blood cell (WBC) levels. The risk of bias for each study was analyzed using the Cochrane’ Risk of Bias’ tool (Table 1).

**Table 1:** Summary of studies included in the meta-analysis.

Leading author (year)y	Number of patients/age range	Study type	Steroid treatment tools	Antibiotics treatment	Judgement of risk of bias
Michael T. (2005)	23/0–15 years	Retrospective	Corticosteroid, dose was not reported	Not reported	Low
Neelam P. (2013)	21/11–59 years	Prospective	Prednisolone 1.5 mg/kg/day for 3 days and 1 mg/kg/day for 3 days	Vancomycin (1 g twice a day or 40 mg/kg/day and Ceftriaxone (1 g twice a day or 100 mg/kg/day)	Low
Brett W. (2014)	31 mean 7.1 years	Prospective	Prednisolone 1 mg/kg/d for 7 days	Ampicillin/sulbactam, Clindamycin, or Vancomycin and Ceftriaxone	High
Lena C. (2017)	28/<18 years (mean 9.3)	Prospective	Dexamethasone 0.3 mg/kg/d for 3 days	Vancomycin, Ampicillin/Sulbactam or Vancomycin, Ceftriaxone, and Clindamycin	Low
Anna B. (2018)	35 children	Retrospective	Prednisolone or methylprednisolone dose ≤1 mg/kg/day	Not reported	Unclear
Joana C. (2019)	122 under 18 years	Retrospective	Not reported	Not reported	Low
Peter J. (2019)	3000/0–18 years	Retrospective cohort study	Not reported	Not reported	Unclear

### Statistical analysis and outcome measurements

The R statistical software (R Foundation for Statistical Computing, Vienna, Austria) was used for the meta-analysis of the selected studies. For continuous variables, meta-analysis was conducted using the Standardized Mean Difference (SMD). The individual mean difference (treatment outcome minus control outcome) was assigned a weight according to the size and standard deviation of each study in order to provide a precise estimate of treatment effect and then summated into a single outcome. For incidence related variables, Odds Ratio (OR) was utilized according to the Mantil-Haenszel method. Heterogeneity was evaluated using the I^2 statistic.

## Results

A total of seven studies among 114 studies were reviewed for the meta-analysis. This analysis included patients admitted to the hospital with a primary diagnosis of periorbital cellulitis or orbital cellulitis made by the attending physician at either admission to the hospital or at discharge. In these studies, they compared the steroid and antibiotics treated groups and antibiotics only treated groups. The results of the bias assessment and study characteristics are described in Table 1. Publication bias was not measured because the number of trials analyzed was insufficient to properly measure with a funnel plot or to perform more advanced regression-based assessments.

**Figure 2:**
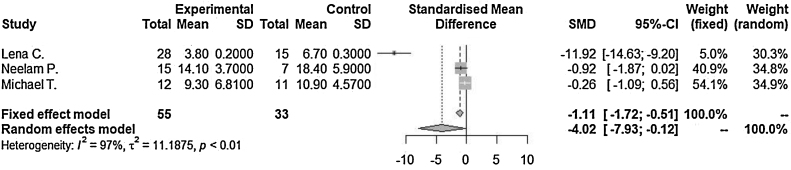
Lengths of hospitalization after admission were compared between in steroid with antibiotics treated group and in only antibiotics treated group. SMD, Standardized mean difference; CI, Confidence interval; SD, Standard deviation.

Lengths of hospitalization after admission as diagnosed as orbital cellulitis (SMD = −4.02 [−7.93; −0.12], *p*-value = 0.04, I^2^ = 96.9%) decreased in steroid with antibiotics treated group compared to antibiotics only treated group significantly (Fig. 2). There was a significant difference between the groups (*p*-value = 0.0435). Significant inter-study heterogeneity was found for this outcome.

**Figure 3:**
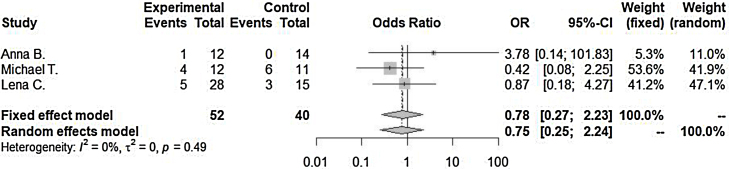
Incidence of surgical drainage were compared between in the steroid with antibiotics treated group and in only antibiotics treated group. SMD, Standardized mean difference; CI, Confidence interval; SD, Standard deviation.

Incidence of surgical drainage (OR = 0.78 [0.27; 2.23], *p*-value = 0.64, I^2^ = 0.0%) was lower in the steroid with antibiotics treated group compared to antibiotics only treated group. However, there were no significant differences between the two groups. Significant inter-study heterogeneity was not found for this outcome (Fig. 3).

There are debates about steroid treatment for orbital cellulitis in several studies. Yen and Yen.^6^ showed that the length of hospitalization between the patients treated with and without intravenous corticosteroids was not significantly different; although there were hospitalizations in the patients treated with systemic corticosteroids. In contrast, Chen et al.^7^ reported that patients who received systemic steroids had significantly shorter hospital stays compared to those of patients who did not receive steroids. The length of hospital stay ranged from 3 to 6 days in the steroid group and 5 to 9 days for the group that did not receive steroids. There were variable data about surgical drainage for extended lesions from orbital cellulitis. For example, in the Yen and Yen^6^ study, 4 of the 12 patients treated with systemic steroids underwent surgical drainage and 6 of the 11 patients treated without steroids underwent surgical drainage. Chen et al.^7^ showed that 18% of patients who received steroids had surgical drainage, but 20% required surgical drainage for their extended lesion, similar to abscess formation in the antibiotics treated group. However, there were not enough data for meta-analysis of orbital cellulitis morbidities: pain, periorbital edema, vision, ptosis, levels o**f** C-reactive protein, and serum WBC levels in our analysis.

## Discussion

Orbital cellulitis attributable to bacterial infection is associated with diffuse edema of the orbital tissues with infiltration by inflammatory cells.^2^ It is a potentially sight- and life- threatening condition similar to permanent visual loss.^2^ This result from panophthalmitis or compressive optic neuropathy attributable to raised intraorbital pressure, toxic optic neuritis, or exposure keratopathy following proptosis.^3^, ^4^

The common management of acute-onset orbital cellulitis is to start a combination of intravenous broad-spectrum antibiotics along with treatment of associated sinusitis; surgical drainage of the abscess is performed if necessary. Although this effectively controls the infective component, inflammation may persist for weeks to months after infection. Nasal mucosa specimens have demonstrated infiltration in the levels of some of these cytokines, IL-1 beta, IL-6, IL-8, and TNF-alpha.^8^

Studies have shown that systemic corticosteroids may be beneficial in treating acute rhinosinusitis, but clinical data on their potential role in orbital cellulitis are scarce. Our study describes a meta-analysis and reviews other papers about the treatment of orbital cellulitis with systemic corticosteroids and antibiotics.

We focused on patients hospitalized with orbital cellulitis treated with systemic steroids during hospitalization. Their demographics, clinical presentation imaging and laboratory findings, treatment protocols, and outcomes were reviewed.

Corticosteroids are known to have a profound anti-inflammatory effect that can reduce the risk of an unfavorable outcome in systemic infections. The beneficial effect of corticosteroids has been proven in many studies on various kinds of inflammatory diseases. In these studies, the authors hypothesized that steroids exert an effect by reducing cerebrospinal fluid, meningeal inflammation, and other neurologic sequelae with no adverse effects.^9^, ^10^, ^11^, ^12^ Corticosteroids are also given in cases of brain abscess to reduce cerebral edema, intracranial tension, and brain herniation.^13^ Further, the role of corticosteroids has been mentioned in literature in cases with septicemia and cavernous sinus thrombosis to suppress excessive inflammation.^14^, ^15^

Although there are many theoretical advantages for the adjunctive use of corticosteroids, meta-analysis of the effect of steroids on orbital cellulitis is lacking. In a review of the English literature, we found several studies evaluating the role of corticosteroids in the acute management of orbital cellulitis. In this retrospective study, the authors concluded that the use of corticosteroids does not adversely affect clinical outcomes and may be beneficial in the treatment of orbital cellulitis.^6^

In Pushker’s study, they supported the fact that the use of corticosteroids as an adjunct to antibiotics has a beneficial effect in acute bacterial orbital cellulitis.^2^ Although complete resolution of infection was seen in all patients, the addition of steroids helped in early resolution of inflammation and improved the overall outcome compared with the no steroids group. They suggested that this combination treatment with antibiotics and steroids may help patients with an inflammatory clinical course, with faster improvements. They analyzed pain scores, periorbital edema, conjunctival chemosis, extraocular motility, and visual acuity. However, disease morbidities did not show statistical significance.

In other cases, in orbital cellulitis caused by community-acquired methicillin-resistant S. aureus, the authors found resolution of cellulitis with the use of intravenous antibiotics and corticosteroids along with surgical drainage of sinuses.^14^ In another report, steroids significantly reduced periocular swelling and led to improved extraocular movements.^15^

On the other hand, a retrospective study found no difference in hospitalization or surgical drainage between patients treated with additional systemic corticosteroids and those treated with antibiotics alone; although there was a slightly reduced hospitalization in the patients treated with intravenous corticosteroids (median, 6.5 days vs. 10 days).^6^

As most studies have used treatment protocols, vancomycin, or broad-spectrum antibiotics, IV ampicillin/sulbactam, clindamycin, or vancomycin and ceftriaxone were given to patients as empirical coverage for both gram-positive and gram-negative organisms.^1^, ^2^, ^3^, ^4^, ^5^, ^6^ None of the patients had adverse effects of steroids, recurrence, or spread of infection. In those studies, the duration of intravenous antibiotics and hospital stay was significantly less in the steroid-treated group, which may help to reduce the cost of care.^1^, ^2^, ^3^, ^4^, ^5^, ^6^

Currently, the use of steroids is still controversial. As other data, 19.7% of patients use systemic steroids for orbital cellulitis.^16^ A previous retrospective study on this issue reported 50% of systemic steroid use in pediatric orbital cellulitis with subperiosteal abscess.^6^ Other retrospective studies did not report the use of steroids.^17^, ^18^, ^19^

A steroid is like a double-edged sword. It decreases edema, inhibits the toxic effect of cytokines and other mediators, helping sinus outflow and hastening the resolution of symptoms.^2^, ^6^, ^20^ However, its use raises concerns about their masking the clinical course and allowing the infection to progress by immunosuppression. Furthermore, in focusing on orbital cellulitis, based on our analysis, we can conclude that the use of steroids for a short period of can shorten the number of hospitalized days. Therefore, we recommend the early use of steroids for a short period with antibiotics before the formation of an orbital abscess.

## Conclusion

Based on our analysis, we can conclude that early use of steroids for a short period can shorten the number of hospitalized days. Therefore, we recommend the early use of steroids for a short period with antibiotics to treat orbital cellulitis. Future research with a larger number of studies should be conducted for sufficient scientific evidence.

## Conflicts of interest

The authors declare no conflicts of interest.
